# Are 25(OH) D concentrations associated with asthma control and pulmonary function test?

**DOI:** 10.22088/cjim.10.4.377

**Published:** 2019

**Authors:** Fatih Uzer, Omer Ozbudak

**Affiliations:** 1 Department of Respiratory Disease, Kastamonu State Hospital, Kastamonu, Turkey; 2 Department of Respiratory Disease, Akdeniz University Hospital, Antalya, Turkey

**Keywords:** Asthma, Deficiency, Exacerbation, Pulmonary function test, Vitamin D

## Abstract

**Background:**

The relationship between vitamin D and asthma is still under investigation. We aimed to evaluate the association between serum vitamin D levels and clinical characteristics of asthma, and the impact of vitamin D deficiency on the clinical manifestations, as them being the issues of debate.

**Methods:**

Patients who were admitted to the outpatient clinics of Chest Diseases Department of Akdeniz University Hospital between January 2014 and December 2014, have been diagnosed as asthma according to the GINA 2014 guidelines were included in this study. Subjects with COPD, bronchiectasis, pneumonia or tuberculosis were excluded. Asthma exacerbation was defined, according to the GINA guidelines, as episodes of progressive shortness of breath, cough, wheezing or chest tightness accompanied by PFT abnormalities such as decreased PEF or FEV1.

**Results:**

A total of 158 patients with mean age of 48.8 years were included in the study. Twenty-seven (17.08%) of the patients demonstrated sufficient vitamin D serum levels (i.e. ≥30 ng/mL), while the remaining 131 (82.92%) patients had vitamin D insufficiency (i.e. <30 ng/mL). When these two patient groups (vitamin D sufficient vs. insufficient) were compared with each other, the patients showing sufficient levels of vitamin D were found to reveal significantly higher FEV1 (L) and FVC (L) values. When the patients were grouped into 4 categories with regard to their serum vitamin D measurements, hospitalization numbers were found to significantly increase with decreasing vitamin D levels.

**Conclusion:**

Vitamin D deficiency was significantly associated with poorer pulmonary functions and higher hospitalization numbers.

Asthma is a chronic and inflammatory airway disease which is under the influence of both genetic and environmental factors. Genetic predisposition is among the most important risk factors for the development of the disease. On the other hand, they are mostly the environmental factors that trigger the exacerbations (1). It is thought that both gene-gene and gene-environment interactions may increase the individuals’ predisposition to asthma. A strong correlation between genetic background and susceptibility to allergic diseases is well established; nonetheless, changes in the global environmental conditions also are accepted to be an important factor which is responsible for the increasing prevalence rates (1, 2). Several theories have been suggested to explain this increasing prevalence of allergy, namely the hygiene and diet hypotheses. The components that the diet hypothesis focuses on can be listed as antioxidants, lipids and other nutrients, food types and dietary patterns, breast milk, probiotics and intestinal microbiota, vitamin D, and maternal diet (3). The relationship between vitamin D and asthma is still under investigation.

Some authors have reported that supplementation with vitamin D might result in asthma; whereas there are others suggesting a negative correlation between asthma and vitamin D (4-6). It has been emphasized in epidemiological studies that vitamin D deficiency is associated with increased asthma symptoms (4). Evidence supporting the significance of vitamin D in immune regulation has been growing. Besides its calcemic effects, the association of vitamin D with several disease conditions has recently been being investigated, and it has been suggested not only to have important roles in the pathogenesis of, but also to be a beneficial treatment option for many. In the NHANES III study, upper respiratory tract infections were found to be more common in patients with low vitamin D levels (7). There are studies reporting that steroid use in severe asthma is associated with vitamin D levels (8). Here in this study, we aimed to evaluate the association between serum vitamin D levels and clinical characteristics of asthma, and the impact of vitamin D deficiency on the clinical manifestations, as them being issues of debate. 

## Methods

Patients who were admitted to the outpatient clinics of Chest Disease Department of Akdeniz University Hospital between January 2014 and December 2014, older than 18 years were included this study. All records were retrospectively screened. The diagnosis of asthma was made according to the Global Initiative for Asthma (GINA) 2014 guideline included in this study (2). Patients with vitamin D levels measured for any reason were included in the study; while those with asthma who were not evaluated for vitamin D levels and those with COPD, bronchiectasis, pneumonia or tuberculosis were excluded. The sample group consisted of patients with asthma and measured vitamin D levels. Age and gender data, pulmonary function tests (PFT), smoking history, bronchodilator drugs, comorbid diseases (cardiovascular diseases, neurological diseases, endocrinological diseases, malignancies etc), number of attacks in 1 year, and hospitalization were recorded. Asthma was diagnosed when patients had symptoms such as episodic breathlessness, wheezing, cough, and chest tightness, and whose spirometry showed bronchial reversibility as 12% and 200 mL from the prebronchodilator value or airway hyperresponsiveness as provocative concentration of a methacholine causing a 20% fall in forced expiratory volume in 1 second (FEV1) was below 25 mg/mL. Asthma exacerbation was defined, again according to the GINA guidelines, as episodes of progressive shortness of breath, cough, wheezing or chest tightness accompanied by PFT abnormalities such as decreased PEF or FEV1. They were treated with steroids and rapid-acting inhaled β-agonists. Pulmonary functions was tested with a spirometer (CareFusion Germany 234 GmbH Yorba Linda, CA 92887). The spirometer was calibrated once daily with a 3-L syringe, according to the operator’s manual. Each patient was tested at least 3 times. The best value was expressed as a percentage of the predicted value and as an absolute value. To assess the vitamin D status of the subjects, serum 25-(OH)-vitamin D measurements were performed using high-performance liquid chromatography. Serum 25-(OH)-vitamin D levels of ≤20 ng/mL were interpreted as vitamin D deficiency, levels of between 21-29 ng/mL as vitamin D insufficiency and levels of ≥30 ng/mL as sufficient. Each patient has performed an asthma control test, as suggested by the GINA 2014 guidelines (2). Statistical analysis was performed with PASW 20 (SPSS / IBM, Chicago, IL, USA). To define sample frequency distribution, mean, standard deviation as descriptive statistics was used. Continuous variables were compared with student’s t test. The chi-square or Fisher exact test was used to compare categorical variables. All p-values are from 2-tailed tests and p-values less than 0.05 were considered statistically significant.

## Results

**Figure 1:**
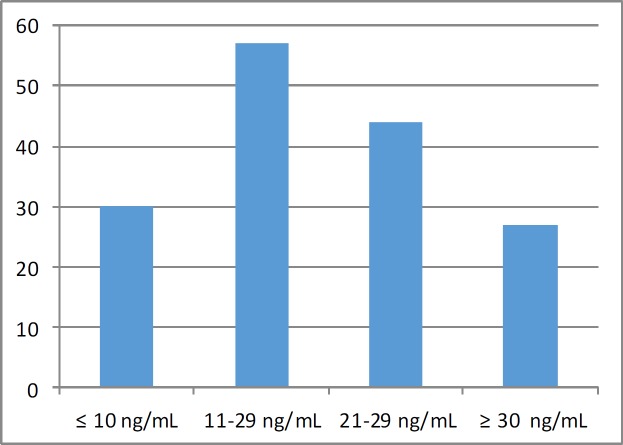
Vitamin D status in the studied patients with asthma

A total of 158 patients with mean age of 48.8 years were included in the study. Table 1 summarizes the fundamental characteristics of the subjects. The most commonly prescribed treatment regimen was the combination of inhaled corticosteroids + long-acting beta-2 agonists + montelukast. Distribution of the patients according to their serum vitamin D levels is shown in figure 1. Twenty-seven (17.08%) of the patients demonstrated sufficient vitamin D serum levels (i.e. ≥30 ng/mL), while the remaining 131 (82.92%) patients had vitamin D insufficiency (i.e. <30 ng/mL). When these two patient groups (vitamin D sufficient vs. insufficient) were compared with each other, the patients showing sufficient levels of vitamin D were found to reveal significantly higher FEV1 (L) and FVC (L) values. All other parameters investigated were comparable between these two groups (Table 2). 

**Table 1:** Basic characteristics of patients

**Characteristics**	**Features**	**Data**
Age (mean±SD)	(years)	48.8±14.5
Sex (%, n)	Male	27.2 (43)
	Female	72.7(115)
Smoking history (%, n)	+ -	20.8 (33) 79.1(125)
25 OH Vitamin (mean±SD)		21.3±14.1
Pulmonary function tests (mean±SD)	FEV_1_(%) FEV_1_ (L) FVC (%) FVC (L)	80.3±22.1 2.4±3.3 82.1±21.7 2.9±3.2
History of exacerbation (previous year) (%, n)	+ -	29.7 (47) 70.2(111)
History of hospitalization (previous year) (%, n)	+ -	7.5 (12) 92.4 146)
Steroid requirement (%, n)	+ -	18.9 (30) 81.0(127)
Asthma control test (mean±SD)		17.7±4.7
Comorbidities (%, n)	+ -	59.4 (94) 40.5 (64)

FEV_1_: forced expiratory volume in 1 second, FVC:forced vital capacity, SD: standard deviation

**Table 2:** Clinical characteristics and pulmonary function tests of patients according to the level of vitamin D

	**Vitamin D** **(<30 ng/mL) (n:131)**	**(≥30 ng/mL) (n:27)**	**p**
FEV_1_ (%) (mean±SD)	79.5±22.5	83.7±19.6	0.578
FEV_1_ (L) (mean±SD)	2.2±0.8	3.7±7.8	0.001
FVC % (mean±SD)	82.3±20.5	81.2±27.3	0.133
FVC (L) (mean±SD)	2.6±1.0	4.1±7.6	0.001
Exacerbation (%,n)	31.3 (41)	22.2 (6)	0.348
Steroid requirement (%,n)	20.8 (27)	11.1 (3)	0.245
Hospitalization (%,n)	7.6 (10)	7.4 (2)	0.968
Smoking history (+) (%,n)	19.1 (25)	29.6 (8)	0.220
Comorbidities (+) (%,n)	58.8 (77)	63.0 (17)	0.687
Asthma Control Test (mean±SD)	17.1±4.7	20.3±3.7	0.001

FEV_1_: forced expiratory volume in 1 second, FVC: forced vital capacity, SD: standard deviation

**Table 3:** The impact of vitamin D levels on asthma severity and pulmonary function tests

	**≤ 10 ng/mL (n:30)**	**11-19 ng/mL** **(n:57)**	**20-29 ng/mL (n:44)**	**≥30 ng/mL (n:27)**	**p**
FEV_1_ (%) (mean±SD)	77.7±26.1	77.0±21.3	84.0±21.1	83.7±19.6	0.315
FEV_1_ (L) (mean±SD)	2.0±0.9	2.1±0.7	2.4±0.9	3.7±7.8	0.180
FVC (%) (mean±SD)	82.2±24.9	80.4±19.4	84.9±18.6	81.2±27.3	0.780
FVC ( L) (mean±SD)	2.5±1.0	2.6±0.8	2.9±1.1	4.1±7.6	0.214
Exacerbation (%,n)	43.3 (13)	35.1 (20)	18.2 (8)	22.2 (6)	0.073
Steroid requirement (%,n)	33.3 (10)	21.4 (12)	11.4 (5)	11.1 (3)	0.074
Hospitalization (%,n)	23.3 (7)	5.3 (3)	0 (0)	7.4 (2)	0.002

FEV1: forced expiratory volume in 1 second, FVC:forced vital capacity, SD: standard deviation

## Discussion

The relationship between vitamin D and asthma is still a subject of investigation. Some previous studies have identified vitamin D as a causative factor for asthma, and some others as a protective agent from the disease. Yet other reports have claimed that vitamin-D deficiency deteriorates asthma control (4, 6, 9). In this work, approximately 70% of our patients showed insufficient vitamin D levels. We have found that vitamin D deficiency results in increased numbers of hospitalization in patients with asthma and that vitamin D insufficiency is associated with poorer pulmonary function test results. 

It is estimated that nearly 1 billion people throughout the world have abnormal vitamin D levels. Presence of vitamin D deficiency has previously been reported in healthy children, young adults, middle-aged individuals and geriatric population (10, 11). A previous study by Freishtat et al. (12) focusing on vitamin D deficiency in asthma, which was performed on young Afro-Americans living in the urban area detected higher prevalence rates of vitamin D deficiency than the control group. Our results revealed a similar vitamin D insufficiency rate of 88.92% among the subjects with asthma. 

Sutherland et al. (13) reported the mean serum vitamin D levels of the adults with asthma as 28.1 ng/mL and found that serum vitamin D level is associated with a better pulmonary function. Our study revealed a mean serum vitamin D level of 21.3 ng/mL in the asthma group. Black et al. (14) reviewed the data from the NHANES III study which had been held in the USA on 14,091 subjects and they found that women had significantly lower mean vitamin D levels than men (28.72 ng/mL vs. 31, 37 ng/mL). The authors suggested that these results might be due to the women’s preference for closed clothing for religious reasons, or their using of sun creams for cosmetic reasons or for protection against skin cancer. There were more females than males included in our study, which may be one reason for our higher vitamin D insufficiency rates when compared with the previous literature. 

Respiratory infections, one condition that is responsible for the flare-ups in asthma patients, are known to be associated with lower vitamin D levels (9). Viruses are the most commonly blamed agents for these exacerbations (15-17). In some studies, patients with high vitamin D levels were found to show reduced needs for hospitalization over the past one year. The same studies also documented that higher levels of vitamin D are associated with a lower requirement for anti-inflammatory drugs (9, 18). In our study, vitamin D deficient patients showed higher hospitalization rates; however, the groups were statistically comparable with regard to need for steroid medication. 

Zosky et al. (19) conducted an animal experiment to reveal whether vitamin D deficiency affects the structure and function of the lungs. In that study, the authors showed that although vitamin D does not alter the somatic growth of the lungs and that the histological structure is almost completely conserved, it leads to decreased lung volumes. The authors concluded that vitamin D deficiency caused deficits in the lung function which was mainly due to alterations in the lung volume, and suggested that that finding might be an explanation of the association between obstructive pulmonary diseases and vitamin D. In recent studies, vitamin D has been thought to have a positive effect on pulmonary functions. In the study by Black et al. (14), serum vitamin D levels were established to be strongly associated with FEV1 and FVC, after standardization of the data for age, gender, ethnicity, body mass index (BMI) and smoking history. Similarly, the results of a Chinese study on 435 asthma patients demonstrated that vitamin D levels were positively correlated with FEV1 (expected %) and FEV1/FVC (expected %) values (20). In our study, there was a positive correlation between vitamin D levels and pulmonary function tests. 

The relationship between vitamin D and asthma severity indicators, such as asthma control, exacerbations, total IgE levels, eosinophil counts and number of hospitalizations for the past 1 year has been investigated. In the study by Ginde et al. (7) on the NHANES III data, which included a total of 18, 883 patients, an association between lower levels of vitamin D and higher rates of upper respiratory tract infections was demonstrated. In that study, it was shown that the association between the increased respiratory tract infection rates and vitamin D levels was more pronounced in the asthma group. It was also demonstrated that asthma attacks were more severe with lower vitamin D levels. The patients with and without sufficient serum vitamin D levels were statistically comparable in terms of asthma exacerbation and hospitalization numbers. However, as vitamin D deficiency deepened, hospitalization rates increased significantly. 

There are number of studies showing that vitamin D deficiency is associated with suboptimal asthma control (21, 22). In our study, vitamin D deficiency did not lead to a significant change in the severity classification of asthma. It was also detected that, in the asthmatic patient group, vitamin D deficiency did not significantly affect asthma control. The mean asthma control test score of the stable asthma patients with vitamin D deficiency did not differ significantly from that of the patients with normal vitamin D levels. According to these findings, it may be concluded that no significant association is present between vitamin D levels and clinical manifestations of asthma. 

Our study had a number of limitations, which should be considered when interpreting the results. These limitations can be summarized as follows: relatively small number of the patients that were included; not completely taking the factors that might affect the vitamin D levels of the individuals into account, such as diet, sun exposure and clothing style; not performing food frequency questionnaire; not analyzing for genetic determinants of vitamin D resistance in the blood samples; not taking seasonality into account; not re-evaluating the subjects with vitamin D deficiency upon completing their replacement therapy.

According to the results of this present study, it was found that serum vitamin D levels and deficiency rates were similar between the asthmatic patients and the control group, but vitamin D deficiency was significantly associated with poorer pulmonary functions and higher hospitalization numbers. Further better planned studies are needed to determine the effect of vitamin D on asthma control.
